# Rare cancer with primary pleural epithelioid hemangioendothelioma diagnosed by thoracoscopic biopsy achieving disease control after 16 months: case report and literature review

**DOI:** 10.3389/fphar.2024.1482154

**Published:** 2024-11-22

**Authors:** Ioannis S. Pateras, Konstantinos Kostopanagiotou, Menelaos G. Samaras, Anna Koumarianou, Mina Koutrouli, Nikolaos Korodimos, Katerina Kampoli, Vasiliki Apollonatou, Effrosyni Manali, Stylianos Loukides, Periklis Tomos, Sotirios Tsiodras, Ioannis G. Panayiotides

**Affiliations:** ^1^ Second Department of Pathology, Medical School, National and Kapodistrian University of Athens, Attikon University Hospital, Athens, Greece; ^2^ Deparmtent of Thoracic Surgery, Attikon University Hospital, Medical School, National and Kapodistrian University of Athens, Athens, Greece; ^3^ Hematology Oncology Unit, Fourth Department of Internal Medicine, Attikon University Hospital, Medical School, National and Kapodistrian University of Athens, Athens, Greece; ^4^ Second Respiratory Medicine Department, Medical School, National and Kapodistrian University of Athens, Attikon University Hospital, Athens, Greece; ^5^ Fourth Department of Internal Medicine, Attikon University Hospital, Medical School, National and Kapodistrian University of Athens, Athens, Greece

**Keywords:** rare cancer, thoracic tumor, paraneoplastic endocrine syndrome, thoracoscopic guided biopsy, pleural epithelioid hemangioendothelioma, management

## Abstract

Epithelioid hemangioendothelioma (EHE) is a rare malignant vascular tumor whose pleural EHE (pEHE) type is even more uncommon, with significant heterogeneity in the clinical behavior as well as challenging diagnosis and treatment decisions. Herein, we present a 74-year-old woman admitted to the hospital with dyspnea, pleural effusion, and refractory euvolemic hyponatremia. Chest computed tomography revealed a large right-side pleural effusion with irregular thickening of the parietal pleura and localized nodularity. Histologic evaluation of the thoracoscopic pleural biopsy tissue was used to confirm the diagnosis of pEHE. Assessment of the volume status suggested that the hyponatremia was attributable to a paraneoplastic endocrine syndrome. Administration of the multitarget tyrosine kinase inhibitor pazopanib helped achieve disease control, with the patient remaining free of symptoms after 16 months of follow-up. This case report adds to the knowledge base of this exceptionally rare condition, highlighting the need for a multidisciplinary approach.

## Introduction

Epithelioid hemangioendothelioma (EHE) is a rare malignant vascular tumor that was initially reported in its pulmonary form as “Intravascular, bronchiolar, and alveolar tumor of the lung” in 1983 (Dail et al., 1983). EHE predominantly affects the soft tissues and skin (30.8%), abdomen (28%), respiratory system (19%), followed by bones and joints (8.6%); its clinical behaviors can be considered to be between hemangioma and angiosarcoma, and its etiology is unknown (Liu and He, 2022). According to the surveillance, epidemiology and end results (SEER) database, the annual incidence rates of EHE are 0.230, 0.272, and 0.164 in Caucasians, African Americans, and other races (including American Indian/Native American/Asian/Pacific Islander) per 1,000,000 persons, respectively (Liu and He, 2022). Pleural epithelioid hemangioendothelioma (pEHE) is even less frequent than other forms of EHE, which makes it an extremely rare condition (Liu and He, 2022; Salijevska et al., 2015; Rezvani et al., 2022). pEHE mainly affects middle-aged men, whereas conventional EHE mainly affects women (Salijevska et al., 2015). Additionally, pEHE has a more aggressive course than other forms of EHE, with the survival ranging from 3 to 24 months (mean of approximately 9.6 months) (Salijevska et al., 2015; Rezvani et al., 2022; Lee et al., 2008).

EHE patients can be asymptomatic or initially exhibit non-specific symptoms, among which the most common are chest pain, persistent cough, and dyspnea, while the least common are weight loss and fever (Salijevska et al., 2015). Chest radiographic findings of pEHE often show pleural thickening, nodularity, and pleural effusions (Crotty et al., 2000). Histopathological evaluations of the thoracoscopic surgical biopsy are often used to confirm the diagnosis. Given the rarity of pEHE, there are no prospective studies available in literature regarding its management.

Herein, we present a patient admitted with dyspnea, pleural effusion, and refractory euvolemic hyponatremia, who was diagnosed with pEHE based on the findings from a thoracoscopic surgical biopsy; the patient was also treated successfully, which adds to the knowledge regarding the diagnosis and management of this rare condition as well as highlights the need for a multidisciplinary approach.

## Case report

A 74-year-old Caucasian woman was evaluated for worsening dyspnea. The patient reported no history of tobacco use. For the 2 months prior to admission, she had been investigated for asymptomatic hyponatremia that was initially attributed to the intake of oral thiazide diuretics for hypertension. Additionally, the patient medical history included diabetes and hypothyroidism, which were treated with oral medications, as well as an episode of deep vein thrombosis of the lower extremity 15 years ago that was attributed to homozygosity for the methylenetetrahydrofolate reductase (*MTHFR*) gene.

Upon auscultation, the right base breath sounds were found to be decreased. The patient was afebrile and hemodynamically stable, with an oxygen saturation value of 97% in room air and a respiratory rate of 18 breaths/min. Assessment of the arterial blood gases with room air inhalation revealed the following values: pO_2_ of 81 mmHg, pCO_2_ of 34 mmHg, pH of 7.46, HCO_3_ of 24 mmol/L, and lactate concentration of 1.4 mmol/L. The blood biochemistry results were as follows: white blood count of 8.91 × 10^3^/μL [normal range (nr): 4.00–11.00 × 10^3^/μL], hemoglobin concentration of 11.5 g/dL (nr: 13.5–17.5 g/dL), creatinine concentration of 1.1 mg/dL (nr: 0.70–1.20 mg/dL), K^+^ concentration of 4.6 mmol/L (nr: 3.5–5.1 mmol/L), Na^+^ concentration of 125 mmol/L (nr: 136–145 mmol/L), spot urine Na^+^ concentration of 110 mmol/L (nr: 40–220 mmol/L), C-reactive protein concentration of 6.32 mg/L (nr: 0–6.00 mg/L), and D-dimer value of 661 ng/mL (nr < 500 ng/L, age adjusted nr <740 ng/L). Despite the low Na^+^ levels, no neurological deficits were detected; however, syndrome of inappropriate antidiuretic hormone secretion (SIADH) promoted by hyponatremia was suspected, which could not be initially confirmed owing to the intake of thiazide diuretics. The patient presented with hypotonic (serum osmolality = 220 mOsm/kg) hyponatremia and elevated urine osmolality (=230 mOsm/kg) in the euvolemic state. Discontinuation of diuretics for 72 h was associated with euvolemic hyponatremia, which favored the diagnosis of SIADH. Volume intake restriction was applied such that the serum sodium level by day 4 was 131 mmol/L (compared to 121 mmol/L upon admission).

**FIGURE 1:**
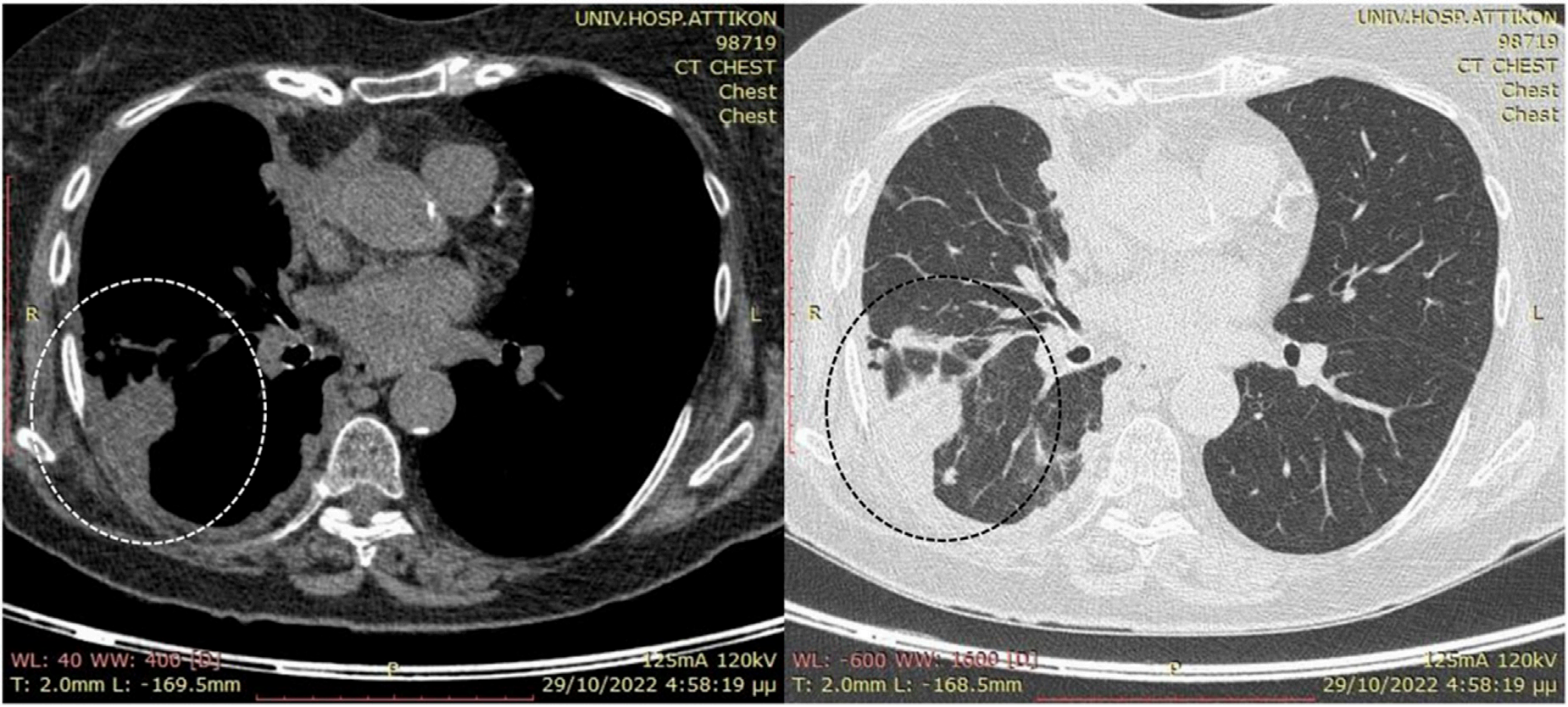
Preoperative computed tomography (CT) scans without intravenous contrast due to suspicion of renal disease. Note the presence of the large right-side pleural effusion (enclosed by the dotted circle) along with localized nodularity at the posterior costal arches and limited pleural fluid in the interlobar fissure after percutaneous drainage.

Computed tomography (CT) scan of the thorax and abdomen revealed a large right-sided pleural effusion with irregularly thickened parietal pleura and localized nodularity at the posterior costal arches (Figure 1). Positron emission tomography (PET)/CT disclosed moderate F-18 fluorodeoxyglucose (FDG) uptake in the pleural thickening (SUVmax 4.4) and lower uptake by the pleural fluid (SUVmax<2) with absent lymphadenopathy or metastatic foci. A diagnostic thoracocentesis produced straw-colored exudate with lymphocytic predominance and the following test results: lactic dehydrogenase of 113 U/L, total proteins of 4.51 g/dL, albumin >2.9 g/dL, glucose value of 110 mg/dL, pH of 7.5, lymphocytes 68%, and polymorphonuclear cells 40%. A percutaneous chest tube was inserted for drainage that initially produced more than 4 L over the first 24 h and subsequently 150–200 mL daily. The pleural fluid cultures were negative for bacteria and mycobacteria; the quantiferon test was negative, and a low pleural adenosine deaminase (ADA) level of 7 IU/L was recorded. The cytology of multiple aspirate samples for malignant cells was found to be negative. The serum immunoglobulin levels were within the normal range, and the brain CT scan was negative for metastatic lesions.

Uniportal thoracoscopic surveillance in the form of video-assisted thoracoscopic surgery (VATS) was performed to monitor the pleural cavity, during which multiple pleural samplings (20 pieces) were collected. The procedure was completed with the insufflation of 8 g of graded sterile talc to achieve pleurodesis and control the recurrent effusions. Macroscopically, the pleural cavity exhibited pathological changes, including pleural granular nodularity and a rich capillary network. Strong adhesions were also present on a large portion of the pleural surface. These findings were not consistent with mesothelioma.

**FIGURE 2:**
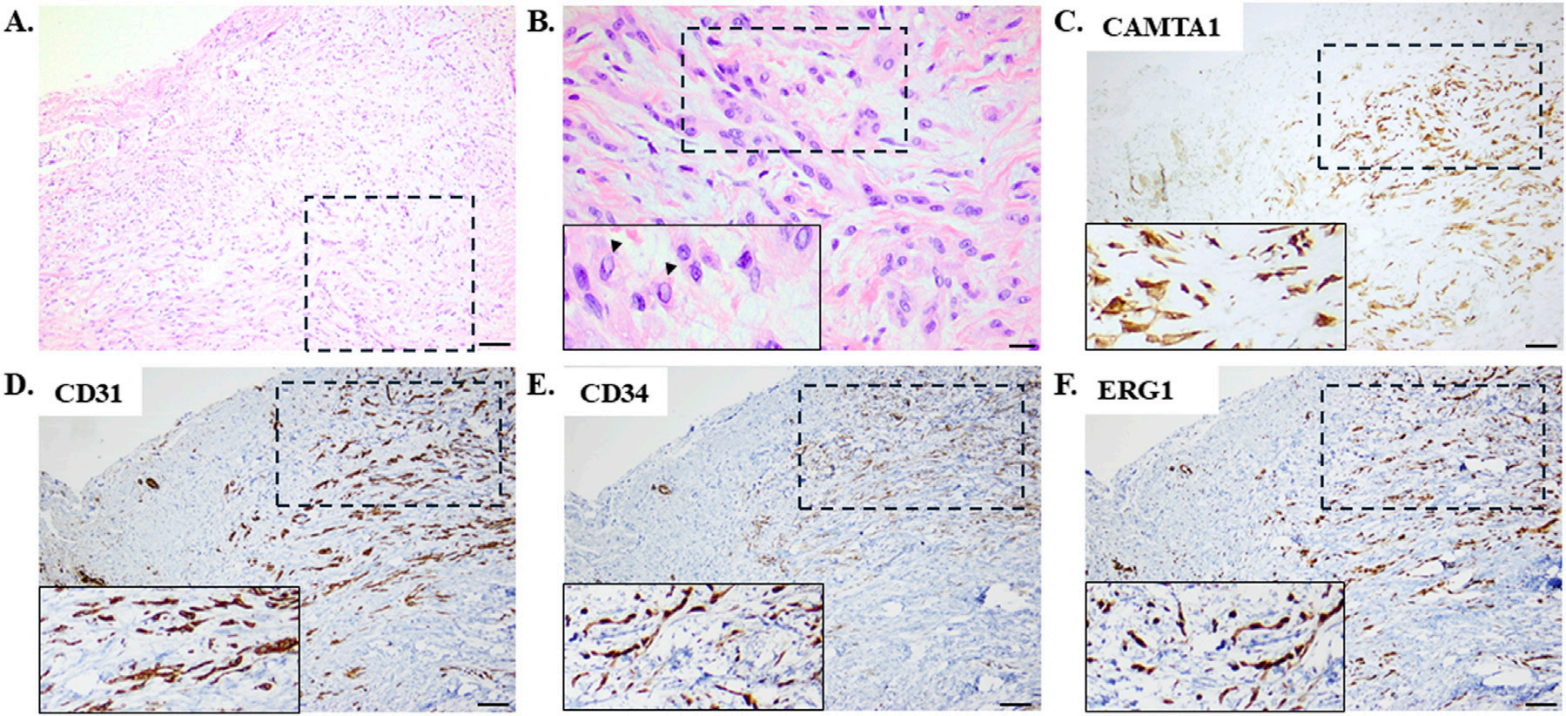
Histological evaluations employing hematoxylin–eosin staining with **(A)** low and **(B)** high magnifications revealed pleural infiltration by epithelioid cells with mild nuclear atypia and moderate eosinophilic cytoplasm with occasional intracytoplasmic vacuoles arranged in a myxohyaline stroma. The arrowheads in the inset image on the bottom left indicate the intracytoplasmic vacuoles. Scale bar: **(A)** 100 μm and **(B)** 20 μm. These representative images (with the dashed rectangles indicating the areas enlarged in the inset images shown on the bottom left) demonstrate neoplastic cells exhibiting diffuse strong nuclear positive immunostaining for calmodulin binding transcription activator 1 (CAMTA1) with intense immunopositivity for the vascular markers CD31, CD34, and ERG. Scale bar: **(C–F)** 100 μm.

Thus far, the differential diagnosis considering the symptoms at presentation along with the pleural effusion was broad and included malignancies, autoimmune diseases, and infections. Histological evaluations revealed infiltration of the pleura by epithelioid cells with mild nuclear atypia and moderate eosinophilic cytoplasm with occasional intracytoplasmic vacuoles that were arranged in strands and cords in a myxohyaline stroma (Figures 2A, B). The neoplastic cells exhibited strong positive immunostaining for calmodulin binding transcription activator 1 (CAMTA1), BRCA1-associated protein 1 (BAP1), and the vascular markers CD31, CD34, and ERG (Figures 2C–F). On the other hand, negative immunostainings were observed for CKAE1/AE3, CK8/18, TTF-1, p40, STAT6, SMA, desmin, calretinin, CK5/6, D2-40, WT1, BerEp4, S100, and SOX10 (Table 1). These histopathological features favored the diagnosis of pEHE.

**TABLE 1:** List of antibodies for which immunostainings were performed on the DAKO platform.

Antibody	Clone (Cat no.)	Supplier	Dilution	pH status	Linker
BAP-1	BSB-109	BioSB	RTU	High	
BerEp4	BerEp4	DAKO	1/100	Low	
Calretinin	DAKCalret-1	DAKO	RTU	High	+
CAMTA1	(NBP1-93620)	Novus Biologicals	1/50	High	
CD31	JC/70A	DAKO	1/50	High	+
CD34	QBEnd10	DAKO	1/75	High	
CKAE1/AE3	AE1/AE3	DAKO	1/150	High	
CK8-18	IVT-2000	Zytomed	1/100	High	
CK5/6	D5/16B4	DAKO	1/50	High	+
Desmin	D33	Zytomed	1/50	High	
D2-40	D2-40	DAKO	1/100	High	
ERG	EP111	DAKO	RTU	High	+ (rabbit)
P40	BC28	Roche	RTU	High	
SMA	1A4	DAKO	1/200	High	
SOX-10	EP268	BioSB	1/200	High	+
STAT6	EP325	Zeta	1/100	High	
S-100	Polyclonal	DAKO	RTU	High	
TTF-1	8G7G3/1	DAKO	1/200	High	+
WT-1	6FH2	DAKO	RTU	High	+

RTU, ready to use.

**FIGURE 3:**
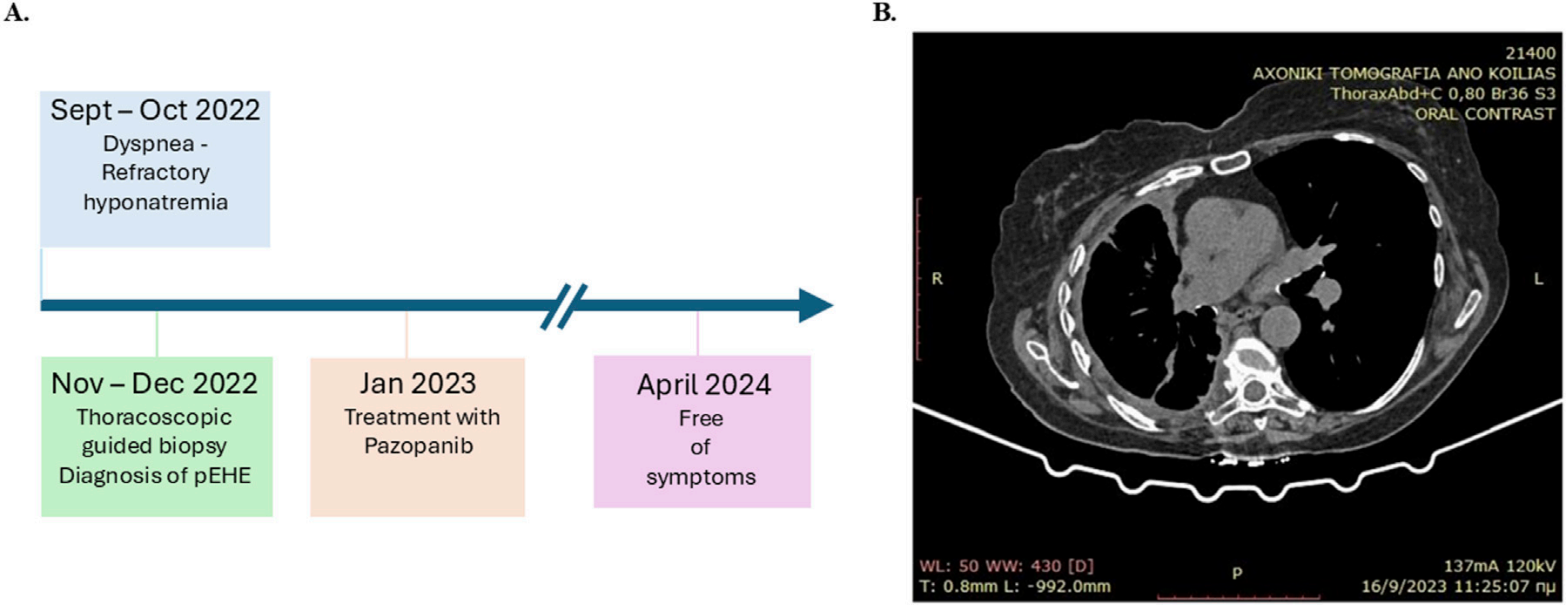
**(A)** Timeline showing the clinical course of the patient for pleural epithelioid hemangioendothelioma (pEHE) and post-diagnosis management. **(B)** Computed tomography (CT) scan obtained 9 months after commencement of first-line therapy.

The postoperative treatment course was uneventful. One month post-surgery, a daily oral dose of 400 mg of pazopanib, which is a multitarget tyrosine kinase inhibitor, was instituted (Figure 3A). After 16 months of follow-up, the patient was found to be free of symptoms with no evidence of metastasis based on biannual CT scans of the abdomen and brain (Figure 3). The extent of pleural disease was also monitored by thoracic CT scans once every 3 months; there were no observed increases in the pleural thickness, no extensions into other areas of the pleural cavity, and no increases in the volume of pleural fluid. Additionally, postoperative monitoring of the sodium levels showed that weekly serum sodium measurements were at the lower end of the normal range (135–137 mmol/L) during the first month. These levels gradually increased above the lower limit of the normal range during the second month and stabilized within the upper levels of the normal range (140–145 mmol/L) in the 3-month assessments thereafter. All other biochemical parameters remained within normal limits throughout the follow-up period. The thoracic CT obtained 1 year later depicted a substantial decrease in the maximum diameter of the affected hemithorax (Figure 3B), possibly owing to pleural scarring post-treatment and the eventual remodeling of the thoracic cage.

## Discussion

pEHE is an extremely rare form of cancer that is frequently unrecognized. Limited case reports in literature have demonstrated heterogeneities in presentation and management, rendering it challenging in terms of diagnosis and treatment (Salijevska et al., 2015). Although no etiology has been established so far, the largest series of pEHE events that included 50 cases reported that eight of the patients had a history of prior exposure to asbestos, while six patients had a history of smoking (Rezvani et al., 2022). In the same study, the majority of patients exhibited chest pain, dyspnea, cough, sputum production, and weight loss, while the uncommon symptoms included the Leser–Trélat sign that is a paraneoplastic syndrome associated with seborrheic keratoses (Crotty et al., 2000). In our reported case, the occurrence of refractory hyponatremia was attributed to SIADH (Pelosof and Gerber, 2010) after ruling out the contributions of thiazide diuretics that are known to commonly induce hyponatremia (Hwang and Kim, 2010) as well as the exclusion of other causes of euvolemic hyponatremia. This is the first case demonstrating SIADH associated with pEHE. In previous reports, one case of hepatic EHE exhibited hypertrophic osteoarthropathy (Kim et al., 2004) and a second case was associated with acute disseminated encephalomyelitis, suggesting a paraneoplastic process (Aktas et al., 2020).

In our case, the indication for VATS biopsy was strongly directed by the PET findings. Cases with low PET avidity of the pleural cavity cannot be considered for surgical biopsy until later in the diagnostic course, thus missing a diagnostic opportunity. One of the advantages of the VATS technique is that it is minimally invasive, which allowed us to explore the entire pleural surface and retrieve targeted biopsies (Cardinale et al., 2017). Recently, a 73-year-old patient presenting with shortness of breath, multiple pulmonary nodules, and pleural thickening has been reported (Pathak and Walker, 2023). Multiple thoracentesis and endobronchial-ultrasound-guided transbronchial needle aspiration could not be used for diagnosis, whereas pleural biopsy after VATS was used to diagnose pEHE (Pathak and Walker, 2023). VATS carries the inherent risks of a surgical procedure, namely, infection, bleeding, cardiovascular and respiratory complications, and the risk of cancer cell dissemination in the pleural cavity in oncological cases, for which appropriate preoperative workup and patient selection can prevent complications (Hanna et al., 2013). One notable contraindication for the VATS biopsy is previous pleurodesis of the ipsilateral pleural cavity, which would prohibit an intrapleural approach.

The molecular hallmarks of conventional EHE include the WW domain containing transcription regulator 1 (WWTR1)–CAMTA1 gene fusion that occurs in the majority of cases (90%), while a small subset of patients present with yes-associated protein 1 (YAP1) and transcription factor E3 (TFE3) gene fusion (Rosenbaum et al., 2020). TAZ, which is the protein encoded by the *WWTR1* gene, and its paralog YAP are part of the Hippo signaling pathway (Seavey et al., 2021). Increasing evidence demonstrates that both WWTR1–CAMTA1 and YAP1–TFE3 genetic alterations play key roles in EHE tumorigenesis (Seavey et al., 2021), suggesting that these genes and their downstream signaling could be therapeutically exploited for EHE. Notably, patients with WWTR1–CAMTA1 gene fusion have less favorable outcomes than those with YAP1–TFE3 gene fusion (Rosenbaum et al., 2020). In our reported case, the strong nuclear immunostaining for CAMTA1 favors the presence of the WWTR1–CAMTA1 fusion gene as it is a surrogate marker for detecting particular translocations (Doyle et al., 2016). Importantly, strong immunopositivity for CAMTA1 is expressed mainly in EHE and not in the EHE cases that mimic other vascular tumors like epithelioid angiosarcoma and epithelioid sarcoma, highlighting its importance as a diagnostic marker for EHE (Doyle et al., 2016). Additionally, the tumor cells in EHE exhibit strong immunostaining for the endothelial markers CD31, CD34, and ERG, retain their BAP1 expressions, and lack expressions for the cytokeratins (including CKAE1/AE3 and CK8/18), TTF-1, p40, GATA3, thus excluding carcinoma; these cells also lack expressions for the mesothelial markers calretinin, CK5/6, WT1, and D2-40, thus excluding mesothelioma (Chou et al., 2024). Another condition that is included in the differential diagnosis is pseudomyogenic hemangioendothelioma; this type is composed of tumor cells with spindle cell morphology (in contrast to the epithelioid morphology), lacks intracytoplasmic vacuoles, and lacks diffused expressions of CAMTA1 and TFE3 even with the expressions of the vascular markers (Jason and Hornick, 2011).

The prognosis of EHE varies according to its anatomical site, with pEHE having worse prognosis than the other forms of EHE, including pulmonary EHE (Rezvani et al., 2022). The common indicators of poor prognosis are the following: patient symptomatic at admission, peripheral lymphadenopathy, presence of metastatic lesions, and pleural effusion (Rezvani et al., 2022). Given the limited experience of the medical community with regard to pEHE, there are no standard therapeutic modalities to date. Surgical removal, along with chemotherapy and/or radiotherapy, has been administered to patients with pEHE, resulting in disease-free survival of more than 14 months (Rezvani et al., 2022). Although upfront surgical resection was reported in a patient with extensive pEHE, the impact of aggressive resection on survival remains to be determined (Hsu et al., 2022). One study showed that a significant proportion of patients receiving anthracycline-based treatments and paclitaxel exhibited partial responses and disease stabilization (Chou et al., 2024). Antitumor activities have been observed with interferon, thalidomide, targeted therapies including multiple tyrosine kinase inhibitors (especially those targeting vascular endothelial growth factor such as pazopanib), and mTOR inhibitors (Chou et al., 2024; Stacchiotti et al., 2021). Moreover, the combination of carboplatin, pemetrexed (an antineoplastic agent involved in folate metabolism), and bevacizumab to block angiogenesis has shown encouraging results in pEHE treatment (Kanemura et al., 2016). Recently, a patient with pEHE was reported to have been treated with trametinib, an inhibitor of mitogen-activated protein kinase (MEK), along with pazopanib to achieve a stable disease course (Khreisat et al., 2024).

## Conclusion

Herein, we describe a patient diagnosed with pEHE associated with a paraneoplastic endocrine syndrome and treated with pazopanib, who showed no signs or symptoms 16 months post-treatment and exceeded the mean prognosis. As pEHE is an extremely rare condition with a high mortality rate, additional reports along with further research efforts are required to advance current understanding and improve patient outcomes. Furthermore, this report highlights the importance of a multidisciplinary approach in the diagnosis and management of pEHE to improve the decision-making processes involved in the treatment of this ultrarare malignancy.

## Data Availability

The original contributions presented in this study are included in the article/Supplementary Material, and any further inquiries may be directed to the corresponding author.

## Abbreviations

BAP1, BRACA1-associated protein 1; CAMTA1, calmodulin binding transcription activator 1; CT, computed tomography; EHE, epithelioid hemangioendothelioma; FDG, F-18 fluorodeoxyglucose; MTHFR, methylenetetrahydrofolate reductase; pEHE, pleural epithelioid hemangioendothelioma; PET, positron emission tomography; SEER, surveillance, epidemiology and end results; SIADH, syndrome of inappropriate antidiuretic hormone secretion; TFE3, transcription factor E3; VATS, video-assisted thoracoscopic surgery; WWTR1, WW domain containing transcription regulator 1; YAP1, yes-associated protein 1.
